# Microbial Eukaryotes Associated With Sediments in Deep-Sea Cold Seeps

**DOI:** 10.3389/fmicb.2021.782004

**Published:** 2021-12-22

**Authors:** Yue Zhang, Ning Huang, Minxiao Wang, Hongbin Liu, Hongmei Jing

**Affiliations:** ^1^CAS Key Laboratory for Experimental Study Under Deep-Sea Extreme Conditions, Institute of Deep-Sea Science and Engineering, Chinese Academy of Sciences, Sanya, China; ^2^Key Laboratory of Marine Ecology and Environmental Sciences, Institute of Oceanology, Chinese Academy of Sciences, Qingdao, China; ^3^Department of Ocean Science, The Hong Kong University of Science and Technology, Hong Kong, Hong Kong SAR, China; ^4^HKUST-CAS Sanya Joint Laboratory of Marine Science Research, Chinese Academy of Sciences, Sanya, China; ^5^Southern Marine Science and Engineering Guangdong Laboratory, Zhuhai, China

**Keywords:** microbial eukaryotes, cold seeps, distribution, diversity, parasitic

## Abstract

Microbial eukaryotes are key components of the marine food web, but their distribution in deep-sea chemosynthetic ecosystems has not been well studied. Here, high-throughput sequencing of the 18S rRNA gene and network analysis were applied to investigate the diversity, distribution and potential relationships between microbial eukaryotes in samples collected from two cold seeps and one trough in the northern South China Sea. SAR (i.e., Stramenopiles, Alveolata, and Rhizaria) was the predominant group in all the samples, and it was highly affiliated to genotypes with potential symbiotic and parasitic strategies identified from other deep-sea extreme environments (e.g., oxygen deficient zones, bathypelagic waters, and hydrothermal vents). Our findings indicated that specialized lineages of deep-sea microbial eukaryotes exist in chemosynthetic cold seeps, where microbial eukaryotes affiliated with parasitic/symbiotic taxa were prevalent in the community. The biogeographic pattern of the total community was best represented by the intermediate operational taxonomic unit (OTU) category, whose relative abundance ranged 0.01–1% within a sample, and the communities of the two cold seeps were distinct from the trough, which suggests that geographical proximity has no critical impact on the distribution of deep-sea microbial eukaryotes. Overall, this study has laid the foundations for future investigations regarding the ecological function and *in situ* trophic relationships of microbial eukaryotes in deep-sea ecosystems.

## Introduction

Marine microbial eukaryotes are fundamental for maintaining the functional stability of marine ecosystems (Caron et al., 2009), and they serve as important links to higher trophic levels as primary producers and consumers (Sherr and Sherr, 2002). In addition, they graze on prokaryotic prey and in this way, they integrate the microbial loop into classical marine food webs (Massana et al., 2004a). Their vast morphological and genetic variability, and consequent physiological diversity enables them to fulfill diverse roles in marine microbial ecosystems (Worden et al., 2015). In recent years, a number of studies have described the diversity and distribution of microbial eukaryotes in various marine habitats (de Vargas et al., 2015; Filker et al., 2018). However, the microbial eukaryotic assemblages that inhabit typical chemosynthetic deep-sea environments such as cold seeps, where the fluids are rich in hydrogen sulfide, methane, and other hydrocarbons, which together generate various chemical and redox gradients, has not been well studied (Takishita et al., 2007).

Microbial eukaryotes with specialized assemblages and trophic status have been recovered from different deep-sea habitats. For example, novel Alveolata and Euglenozoa species with parasitic trophic status have been recovered as dominant groups from three abyssal plains in the southeastern Atlantic Ocean (Takishita et al., 2010). In addition, novel Kinetoplastids have been recovered from a hypersaline deep-sea basin in the eastern region of the Mediterranean (Edgcomb V. P. et al., 2011). It has also been shown that sulfidic and anoxic zones in the deep-sea harbor a great diversity of unclassified ciliates (Coyne et al., 2013) and Stramenopiles (Wylezich and Jürgens, 2011) with unknown ecological functions, and hydrothermal vents has been suggested as oases for parasitic protists (Moreira and López-Garcia, 2003). Regarding cold seep sediments, fungi, and symbiotic ciliates have been found to be major microbial eukaryotes in microbial mats (Takishita et al., 2010) and anoxic layers (Orsi et al., 2012), whereas Radiolarian often predominate in the anaerobic sediment-water interface (Kouduka et al., 2017). Diversity, distribution (Pasulka et al., 2016) and the potential trophic relationships of microbial eukaryotes in this environment has not been explored. Considering cold seeps to be typically chemosynthetic ecosystems, it is reasonable to assume that specialized microbial eukaryotic assemblages with possible parasitic or symbiotic trophic status exist in the microbial niches of cold seep sediments.

Various cold seeps have been detected along the northern slope of the South China Sea (SCS) (Feng and Chen, 2015). Among them, the Haima cold seep (located in the southern part of the Qiongdongnan Basin), has exhibited a decline in activity in recent years (Liang et al., 2017). In contrast, the Formosa Ridge (or Site F) cold seep (located offshore from southwestern Taiwan), is currently active (Feng and Chen, 2015). By far, most of the microbial studies conducted in these two cold seeps have focused on prokaryotes (Niu et al., 2017; Ling et al., 2020), whereas microbial eukaryotes have largely been overlooked (Takishita et al., 2007, 2010). In cold seep environments, a combination of the *in situ* reductive conditions and availability of oxygen along the vertical sediment layers, might affect the distribution of eukaryotic groups (Mazouni et al., 1996; Neat et al., 2018). Therefore, a comprehensive analysis of the microbial eukaryotes that inhabit the stratified sediments in different cold seeps is necessary.

In this study, push core sediment samples from the Site F and Haima cold seeps were collected. Samples were also collected from the Xisha Trough as controls. This trough is located in the northwestern part of the SCS between the two cold seeps and it has a sedimentary geology favorable for the formation and accumulation of gas hydrates (Yang et al., 2007). At each sampling station, the top three sections with an interval of 2 cm for each sediment core, were retrieved. With this sampling strategy, the diversity, composition, and distribution of microbial eukaryotes were compared in the sediments of deep-sea cold seeps subjected to different reductive conditions along geographical scales ranging from just a few centimeters to hundreds of kilometers.

## Materials and Methods

### Sample Collection and Nutrient Analysis

**FIGURE 1:**
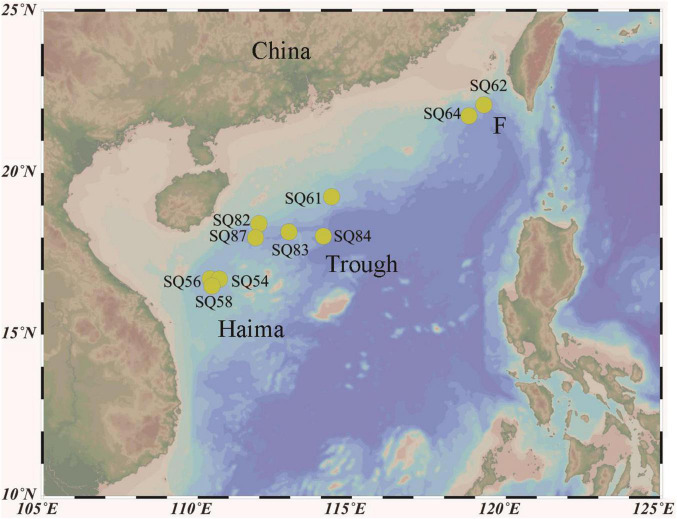
Map of the sampling stations in the northern South China Sea.

Push core sediment samples were collected from the Haima (16°43′N, 110°28′E; SQ_54, SQ_56, and SQ_58), and Site F (22°6′N, 119°17′E; SQ_62 and SQ_64) cold seeps as well as from the Xisha Trough (18°18′N, 114°08′E; SQ_61, SQ_82, SQ_83, SQ_84, and SQ_87) in the SCS during cruise TS07 on R/V “Tan Suo Yi Hao” in June 2018 (Figure 1). *In situ* hydrographical parameters (i.e., depth and location) were recorded during sampling using the manned submersible, SHENHAI YONGSHI. Sediment push core samples were collected underwater by HOV using a polyvinyl chloride pipe. Once on board, the sediment cores were immediately sliced into of 2 cm in thickness with sterilized shovel, and stored at −80°C. The top three layers, each of 2 cm in length [i.e., 0–2 cm (surface), 2–4 cm (middle), and 4–6 cm (deepest)], were acquired for further analysis. A sediment properties analysis [i.e., nitrate, ammonia, total carbon (TC), and total nitrogen (TN)] was conducted with approximately 5 g of frozen sediment at the Institute of Mountain Hazards and Environment, Chinese Academy of Sciences (Chengdu, Sichuan, China), according to Wang et al. (2016). In brief, nitrate and ammonia were detected after 1 M HCl treatment followed by analysis with a colorimetric auto-analyzer (SEAL Analytical AutoAnalyzer 3, Germany). The TC and TN concentrations were determined by over-drying the sediments at 105°C and then using an element analyzer (Elementar vario Macro cube, Germany). Each parameter was measured in triplicates and values with standard error < 1% were treated as valid.

### DNA Extraction, PCR Amplification and Sequencing

The sediments were centrifuged to remove any pore water, and then total DNA was extracted from the 0–2 cm (surface), 2–4 cm (middle), and 4–6 cm (deepest) sediment layers with the PowerSoil DNA Isolation Kit (MO BIO Laboratories, Inc., Carlsbad, United States), according to the manufacturer’s protocol. The DNA was quantified with Qbuit^®^ 2.0 (Life Technologies, United States), and the quality was checked via gel electrophoresis.

DNA was amplified using the FastStart High Fidelity PCR system (Roche) with the following universal primers: TAReuk454FWD1 [5′-CCAGCA(G/C)C(C/T)G CGGTAATTCC-3′] and REV3 [5′-ACTTTCGTTCTTGA T(C/T)(A/G)A-3′] (Stoeck et al., 2010), to target the V4 domains of the 18S rRNA gene. The polymerase chain reaction (PCR) was performed with an initial denaturation step of 95°C for 3 min, followed by 32 cycles of 95°C for 30 s, 55°C for 30 s, and 72°C for 1 min, after which there was a final extension step of 72°C for 5 min. A negative control comprising double-distilled water alone, was also included during amplification to test for reagent contamination. The paired-end sequencing of the amplicons was performed with an Illumina HiSeq PE250 sequencer (Novogene Co., Ltd.)^1^.

### Quantitative PCR

The abundance of the 18S rRNA gene was quantified using the StepOnePlus quantitative PCR (qPCR) system (Applied Biosystems Inc., Carlsbad, CA, United States). Each qPCR reaction comprised 10 μL 2 × SYBR^®^ Premix Ex Taq™ II (Takara Bio Inc., Shiga, Japan), 0.3 μM euk345f/Euk499r primer (Zhu et al., 2005), 2 μL DNA as the template, 0.4 μL ROX reference dye, and water to a total of 20 μL. The qPCR reactions and calibrations were performed following a protocol described previously (Zhu et al., 2005). As a positive control, a linear plasmid was used, which was constructed using the amplified PCR products and a TOPO-TA vector cloning kit (Invitrogen). Standard curve was constructed based on qPCR with a serial dilution of the linear plasmid to get a relationship between the Ct value and the gene copies. Triplicate qPCR reactions were performed for each sample with efficiencies of 94.51%, and the Ct values were calculated as gene copies against the standard curve. The gene abundance per sample was obtained by multiplying the elution volume and dividing the weight of sediment during DNA extraction.

### Bioinformatics Analysis

After sequencing, barcoded and low-quality sequences were removed using QIIME v1.9.1 with default parameters (Caporaso et al., 2010). Chimeras were detected and removed with UCHIME v6.1 against the PR^2^ database (Guillou et al., 2013), and reads that presented as a single copy (i.e., singletons) were manually removed. The remaining reads were then clustered into Operational Taxonomic Units (OTUs) at 97% sequence similarity. Taxonomic assignment of OTUs that were not affiliated with microbial eukaryotes (including bacteria and archaea, as well as metazoan and plastidial sequences), as determined by the PR^2^ database (Guillou et al., 2013), were also removed (Caporaso et al., 2010). A filtered OTU table of each sample was generated with QIIME 1.9.1. The Shannon and Simpson index were then calculated, with 97% sequence similarity as the cutoff value. The distribution of different clades affiliated to the parasitic Syndiniales was performed using “pheatmap” packages in R version 3.5.3.

Three categories of OTUs were defined based on their relative abundance, i.e., abundant (OTUs ≥ 1% within a sample), intermediate (0.01 < OTUs < 1% within a sample) and rare (<0.01% within a sample). Dichotomy diagrams were prepared and graphically edited in Cytoscape v3.8.0 (Shannon et al., 2003) using the “edge-weighted spring embedded” layout with weights. In addition, the community structure and relative abundance of microbial eukaryotes were visualized via bar and bubble charts prepared using the “ggplot2” packages in R v3.5.3.

### Network Analysis

To explore the co-occurrence patterns between taxa, a network analysis was conducted by calculating the correlations. A similarity matrix was firstly generated by inputting a typical OTU matrix file, and then the correlation matrix, *R*-value and *p*-value were calculated using corr. test in the “psych” package of R v3.5.3. The threshold value was calculated to reflect the interaction between species, and value within the range of occor.*p* > 0.01| abs (occor.r) < 0.6 was converted to 0 to obtain a CSV file, which was input into Gephi v0.9.2 to construct the networks with Spearman’s ρ > 0.6 and FDR-adjusted *p* < 0.05 (Bastian et al., 2009). In addition, the phylogenetic affiliation of the most abundant 21 OTUs identified from the major modules of the network analysis were determined by constructing two Maximum Likelihood (ML) trees using MEGA6 (Tamura et al., 2013) with the General Time Reversible model and K2+G method for 1,000 bootstrap iterations.

### Statistical Analysis

Non-linear multidimensional scaling (nMDS), based on the Bray-Curtis similarity index, was calculated with PRIMER 5 (Plymouth Marine Laboratory, West Hoe, Plymouth, United Kingdom) to show the similarity among the different samples. In addition, an analysis of similarities (ANOSIM) based on the OTU relative abundance was conducted with Paleontological Statistics (PAST) v3 (Hammer et al., 2001) to test whether there was a significant difference in the microbial community among the various sampling sites. Furthermore, the similarity percentage analysis (SIMPER) test was performed to show which group of organisms was responsible for the dissimilarity observed in community composition among different samples using Paleontological Statistics (PAST) v3 (Hammer et al., 2001).

### Availability of Data

All the 18S rRNA genes obtained from this study have been deposited in the National Center for Biotechnology Information (NCBI) Sequence Read Archive (SRA) under the accession number PRJNA742151.

## Results

### Hydrographic Conditions

The total nitrogen content was higher in Haima (1.61–2.17 mg/kg, mean = 1.91) than in either Site F (1.21–1.45 mg/kg, mean = 1.27) or Xisha Trough (1.06–1.65 mg/kg, mean = 1.47) (Table 1). In contrast, the highest TC and C/N were found at Stn. SQ61-1 (TC: 34.52–52.37 mg/kg, mean = 45.67; C/N: 24.66–47.29 mg/kg, mean = 38.41), and both were significantly higher than in the other sites (*p* < 0.01). In general, the highest concentration of NH_4_^+^ was in the 7 out of 10 surface layers (0–2 cm). The concentration of NO_3_^–^ was much lower than that of NH_4_^+^ but it was also highest in the shallow sediment depths, i.e., in the surface layer (0–2 cm) in Haima (17.02–42.88 mg/kg, mean = 31.8 mg/kg) and Site F (8.61–20.31 mg/kg, mean = 14.46 mg/kg) and in the middle (2–4 cm) layer in Xisha Trough (2.60–42.40 mg/kg, mean = 11.59 mg/kg).

**TABLE 1:** The environmental parameters, sequencing information and diversity index of sediments collected from the cold seep and trough sampling sites in the northern South China Sea.

	Station	TN (mg/g)	TC (mg/g)	C/N	NO_3_^–^ (mg/kg)	NH_4_^+^ (mg/kg)	Original reads	Quality reads	Reads (exclude metazoa)	OTUs (97%)	Shannon	Simpson
Haima	SQ54-1	2.11	12.36	5.86	17.02	148.95	81,018	20,680	17,963	385	6.90	0.98
	SQ54-2	2.17	12.32	5.68	15.50	123.85	64,489	16,553	15,924	410	7.04	0.98
	SQ54-3	2.05	12.35	6.02	14.13	105.53	80,514	29,456	28,818	495	6.98	0.98
	SQ56-1	1.70	11.26	6.62	42.88	113.99	75,450	35,973	16,623	434	5.24	0.85
	SQ56-2	1.72	11.15	6.48	42.97	105.22	78,789	21,757	9,522	268	4.08	0.73
	SQ56-3	1.61	10.64	6.61	33.81	117.64	78,479	34,913	20,930	329	5.22	0.91
	SQ58-1	1.85	11.19	6.05	35.50	145.17	84,374	35,397	28,681	329	5.94	0.95
	SQ58-2	2.04	11.84	5.80	10.84	143.60	91,575	16,949	7,836	201	5.13	0.92
	SQ58-3	1.97	11.36	5.77	3.96	126.60	88,006	38,384	30,128	425	6.29	0.97
	SQ62-1	1.21	6.25	5.17	8.61	104.25	72,579	32,184	19,672	362	5.54	0.91
F	SQ62-2	1.29	6.21	4.81	8.77	97.20	86,281	40,573	15,057	243	3.46	0.64
	SQ62-3	1.22	6.13	5.02	7.35	101.29	81,389	29,302	23,220	212	4.91	0.91
	SQ64-1	1.45	7.72	5.32	20.31	141.46	85,535	35,202	20,142	457	5.91	0.94
	SQ64-2	1.21	7.36	6.08	17.88	120.28	78,694	37,004	29,602	466	6.00	0.95
	SQ64-3	1.24	7.29	5.88	8.16	79.10	74,327	31,212	30,646	464	6.17	0.95
Trough	SQ61-1	1.21	52.37	43.28	4.39	108.06	75,461	32,862	22,712	531	6.99	0.98
	SQ61-2	1.06	50.13	47.29	4.59	111.64	81,100	33,518	30,130	394	6.40	0.97
	SQ61-3	1.40	34.52	24.66	5.73	97.56	73,064	40,154	10,850	298	3.78	0.75
	SQ82-1	1.65	9.95	6.03	4.79	99.87	89,191	38,647	10,664	321	2.99	0.53
	SQ82-2	1.55	9.93	6.41	5.85	114.64	81,336	41,895	1,786	128	0.73	0.12
	SQ82-3	1.40	10.21	7.29	4.36	95.63	75,205	41,127	10,759	286	2.68	0.49
	SQ83-1	1.64	9.62	5.87	38.44	157.84	73,723	21,080	19,350	312	6.10	0.96
	SQ83-2	1.64	8.37	5.10	42.40	136.92	79,861	21,861	19,040	253	6.17	0.97
	SQ83-3	1.44	7.58	5.26	8.25	118.18	85,079	29,275	25,682	246	5.68	0.95
	SQ84-1	1.26	7.00	5.56	2.05	144.92	80,223	33,924	33,542	317	5.36	0.90
	SQ84-2	1.45	6.29	4.34	2.60	120.71	89,945	43,867	43,074	480	6.34	0.96
	SQ84-3	1.56	7.90	5.06	1.82	103.04	89,591	39,793	38,011	465	6.55	0.97
	SQ87-1	1.63	9.88	6.06	3.06	129.84	76,638	34,852	20,483	420	5.74	0.91
	SQ87-2	1.57	8.29	5.28	2.53	118.69	90,567	41,678	40,737	429	6.44	0.97
	SQ87-3	1.59	9.51	5.98	2.47	99.53	87,630	38,236	37,011	411	6.52	0.97

*In the station column, numbers 1, 2, and 3 represent the 0–2, 2–4, and 4–6 cm sediment layers, respectively.*

### Vertical Profile of Diversity and Abundance

**FIGURE 2:**
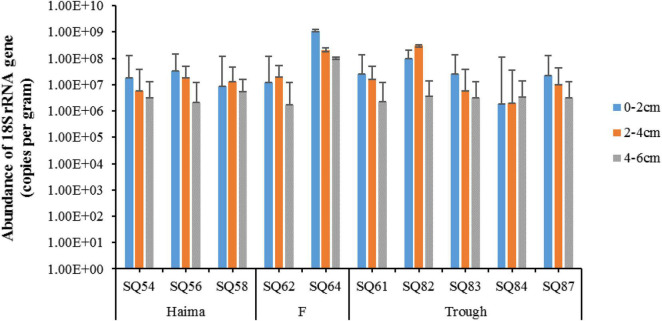
The 18S rRNA gene abundance of microbial eukaryotes in the sediment samples of the northern South China Sea. The error bars represent standard deviation of triplicate reactions for each sample.

In total, 988,126 sequences and 3,969 OTUs were generated from quality reads using 97% as the clustering threshold (Table 1). The maximum number of OTUs was found at Stn. SQ64-1. Along the vertical profile, the highest diversity (Shannon and Simpson index) of microbial eukaryotes occurred at Stn. SQ54-2. In addition, the gene abundance of microbial eukaryotes was determined by qPCR with primer set of euk345f/Euk499r, which selectively amplify Prasinophyceae, Trebouxiophyceae, Chlorophyceae, Cryptophyceae, Bacillariophyceae, Chrysophyceae, Dictyochophyceae, and Dinophyceae, has been wildly applied in different studies on protists (Zou et al., 2020) or microbial eukaryotes (Gong et al., 2020), and metazoans should be excluded. In general, the gene abundance of the microbial eukaryotes was significantly lower in the deepest layer (AMOSIM, *p* < 0.01), apart from Stn. SQ_84 (Figure 2). Among the three sampling sites, the highest gene abundance was detected at Site F (2.21–911.2 × 10^6^ copies/g, mean = 2.00 × 10^8^ copies/g).

### Community Composition of the Microbial Eukaryotes

**FIGURE 3:**
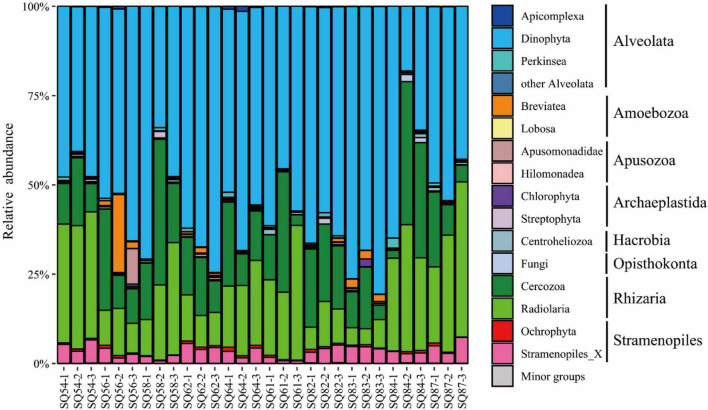
Community structure of the microbial eukaryotes (excluding metazoa) in the sediment samples of the northern South China Sea at the super-group level. The numbers 1, 2, and 3 represent the 0–2, 2–4, and 4–6 cm sediment layers, respectively.

The SAR (i.e., Stramenopiles, Alveolata, and Rhizaria) super-group dominated in all three habitats (Figure 3). In contrast, the proportion of other super-groups (for example, Opisthokonta, Amoebozoa, and Apusozoa), together accounted for less than 10% in each sample. Regarding the Alveolata group, Dinophyta was the dominant assemblage in each sample, whereas Radiolaria and Cercozoa were the main components of the Rhizaria group, and Ochrophyta was the predominant group of the Stramenopiles. In contrast to the distribution of Rhizaria, the relative abundance of Alveolata at Stns. SQ84 and SQ87 was about one-third less than that at other sampling stations. In addition, the relative abundance of Alveolata was the lowest in the 7 out of 8 middle layers (2–4 cm) in both Haima and Xisha Trough, and in the surface layer (0–2 cm) in each station of Site F. In contrast, higher abundance of Rhizaria was detected in the 7 out of 10 middle layers (2–4 cm), and Stramenopiles were most abundant in the 6 out of 10 deepest layers (4–6 cm). SIMPER analysis suggested the “indicative OTUs” that contributed ≥ 1% dissimilarity between the cold seeps and trough were belonged to Alveolata (mainly Dino-Group-I and Dino-Group-II) and Rhizaria (mainly Cercozoa and Spumellarida).

Sequences belonging to metazoa accounted for 1.9–75.1% in each sample (Supplementary Figure 1A). These consisted mainly of Hemichordata (35.9%), Annelida (24.8%), Nematoda (12.4%), Nemertea (9.8%), and Platyhelminthes (8.8%) (Supplementary Figure 1B). The Hemichordata and Nematoda accounted for higher proportions in both the cold seeps, whereas the Annelida and Nemertea were more abundant in the trough. In addition, Nematoda species were more highly abundant in the surface layer in the cold seeps and trough, whereas Hemichordata species were more abundant in the deepest layer.

The distribution of different clades affiliated to parasitic Syndiniales in all the samples was exhibited in the heatmap (Supplementary Figure 2). Dino-Group-I and II were highly represented in current study, with 8 and 25 clades identified, respectively. In the Dino-Group-I, clade-1 and 2 accounted for higher proportions than other clades, while clade-10/11 and 15 were major clades in the Dino-Group-II. Dino-Group-III and IV (mainly Hematodinium and Syndinium) were also present with much less abundance in all samples.

Pearson correlation coefficient analysis showed that the TC and C/N ratio had significant negative correlation with the relative abundance of Stramenopiles (*p* < 0.05). The concentration of NO_3_^–^ explained most of the total variance of the microbial eukaryotic community in the cold seeps, where it was especially negative correlation with the Radiolaria (*p* < 0.01), and positively correlated with Breviatea (*p* < 0.01) and Chlorophyta (*p* < 0.05). In addition, the concentration of NH_4_^+^ exhibited a significant positive correlation with Perkinsea (*p* < 0.05) (Table 2).

**TABLE 2:** The Pearson correlation coefficients indicating the relationship between various microbial eukaryotes (with relative abundance > 2%), and different environmental parameters in the sediment samples collected from the northern South China Sea.

	Total N	Total C	C/N	NO_3_^–^	NH_4_^+^
Dinophyta	–0.33	–0.07	–0.04	0.29	0.02
Radiolaria	0.26	0.15	0.09	−0.48**	–0.07
Cercozoa	0.11	0.07	0.09	–0.08	0.09
Stramenopiles	0.12	−0.46*	−0.45*	–0.02	–0.09
Breviatea	0.09	–0.06	–0.08	0.49**	–0.10
Perkinsea	–0.07	–0.12	–0.10	–0.17	0.38*
Ochrophyta	–0.13	–0.23	–0.20	0.15	–0.19
Apusomonadidae	0.02	–0.04	–0.05	0.27	–0.01
Centroheliozoa	0.08	0.31	0.27	–0.15	0.03
Streptophyta	0.18	–0.12	–0.15	–0.22	–0.10
Fungi	0.03	–0.20	–0.18	–0.24	–0.05
Chlorophyta	–0.03	–0.12	–0.10	0.45*	0.16
Apicomplexa	–0.19	–0.18	–0.15	0.26	–0.01

*p < 0.05; **p < 0.01.

### Community Similarity

**FIGURE 4:**
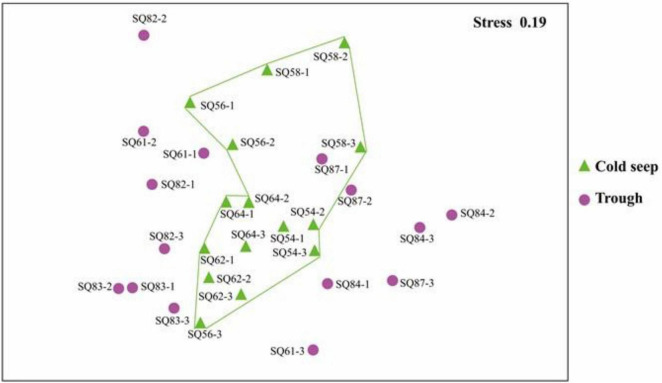
nMDS plot based on the total OTUs in the sediment samples collected in the northern South China Sea. The numbers 1, 2, and 3 represent the 0–2, 2–4, and 4–6 cm sediment layers, respectively.

NMDS analysis showed that (excluding metazoan related OTUs), samples from cold seeps were clustered together, and they were distinct from those in the trough regions, whereas no clear clustering was observed based on sedimentary depth (Figure 4).

**FIGURE 5:**
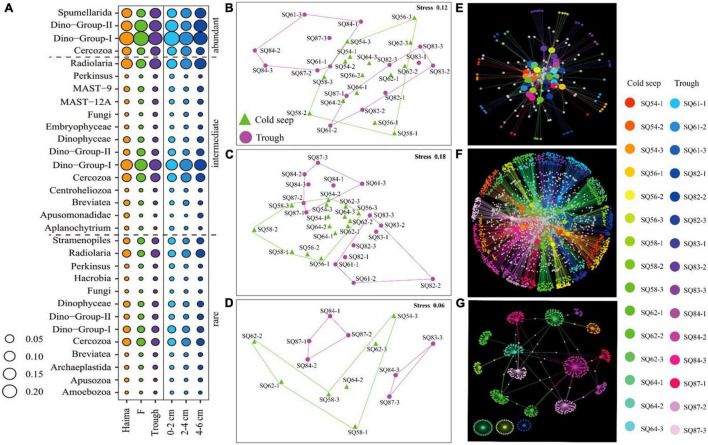
Community structure **(A)** and nMDS plots **(B–D)** based on the relative abundance of the respective abundant **(B)**, intermediate **(C)** and rare **(D)** OTU categories. The size of bubbles reflects the proportion of each taxon. Network diagrams **(E–G)** based on the relative abundance of the respective abundant **(E)**, intermediate **(F)** and rare OTUs **(G)**; the large nodes represent the samples, and the small nodes represent the OTUs. The white notes represent OTUs that were shared between samples. In **(B–G)**, the numbers 1, 2, and 3 represent the 0–2, 2–4, and 4–6 cm sediment layers, respectively.

The abundant, intermediate, and rare categories were further analyzed, which accounted for 60.55, 39.21, and 0.243% of the total OTUs, respectively (Table 3). Dino-Group-I and II were dominant in the abundant category, whereas Cercozoa was a major group in the rare category (Figure 5A). In addition, a lower level of segregation was found in the three habitats in the abundant category (Figure 5B), and a clear clustering of the cold seeps distinct from the trough was only observed in the intermediate category (Figure 5C). In addition, the deepest layer seemed to be distinct from the upper layers, especially in the trough (Figure 5D). A dichotomy network diagram was constructed to illustrate the similarity of community structure among the three OTU categories. The highest similarity among the samples was observed in the abundant category (Figure 5E), whereas the maximum number of total and shared OTUs was found in the intermediate category (Figure 5F), and unique OTUs to each site occupied the rare category (Figure 5G).

**TABLE 3:** Sequencing information of the abundant, intermediate, and rare OTUs of sediment samples collected from the South China Sea.

Sample	(>1%)			(0.01–1%)			(<0.01%)		
	OTUs	Read	Read (%)	OTUs	Read	Read (%)	OTUs	Reads	Read (%)
SQ54-1	26	10,376	57.76	296	7,587	42.24	0	0	0.00
SQ54-2	22	7,975	50.08	354	7,949	49.92	0	0	0.00
SQ54-3	23	15,514	53.83	402	13,238	45.94	33	66	0.23
SQ56-1	22	8,236	49.55	327	8,387	50.45	0	0	0.00
SQ56-2	24	5,949	62.48	201	3,573	37.52	0	0	0.00
SQ56-3	21	14,292	68.28	238	6,582	31.45	28	56	0.27
SQ58-1	21	20,040	69.87	216	8,587	29.94	27	54	0.19
SQ58-2	24	5,732	73.15	121	2,104	26.85	0	0	0.00
SQ58-3	22	18,214	60.46	317	11,781	39.10	54	133	0.44
SQ62-1	17	10,249	50.88	346	9,825	48.78	34	68	0.34
SQ62-2	16	17,265	58.32	383	12,263	41.43	37	74	0.25
SQ62-3	17	18,092	59.04	352	12,391	40.43	64	163	0.53
SQ64-1	19	11,998	52.83	360	10,650	46.89	32	64	0.28
SQ64-2	21	18,541	61.54	273	11,478	38.09	46	111	0.37
SQ64-3	23	5,877	54.17	238	4,973	45.83	0	0	0.00
SQ61-1	21	12,399	63.03	280	7,273	36.97	0	0	0.00
SQ61-2	24	10,127	67.26	178	4,930	32.74	0	0	0.00
SQ61-3	16	16,696	71.90	159	6,494	27.97	15	30	0.13
SQ82-1	19	6,009	56.35	240	4,655	43.65	0	0	0.00
SQ82-2	25	1,281	71.72	70	505	28.28	0	0	0.00
SQ82-3	22	6,858	63.74	232	3,901	36.26	0	0	0.00
SQ83-1	19	12,231	63.21	259	7,119	36.79	0	0	0.00
SQ83-2	24	12,470	65.49	198	6,570	34.51	0	0	0.00
SQ83-3	18	18,175	70.77	194	7,483	29.14	12	24	0.09
SQ84-1	17	23,146	69.01	251	10,322	30.77	31	74	0.22
SQ84-2	21	25,995	60.35	353	16,837	39.09	83	242	0.56
SQ84-3	15	20,097	52.87	384	17,793	46.81	48	121	0.32
SQ87-1	20	10,941	53.42	318	9,482	46.29	30	60	0.29
SQ87-2	21	24,648	60.51	323	15,908	39.05	62	181	0.44
SQ87-3	20	21,453	57.96	329	15,458	41.77	40	100	0.27

*Singletons and sequences associated with metazoan taxa were removed. In column 1, the numbers 1, 2, and 3 represent the 0–2, 2–4, and 4–6 cm sediment layers, respectively.*

### Co-occurrence Network

**FIGURE 6:**
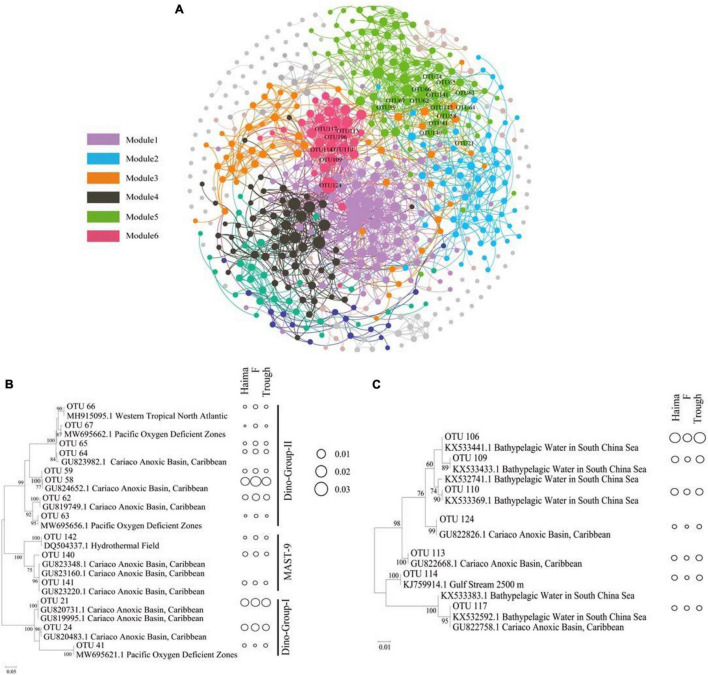
Networks of co-occurring microbial eukaryotic taxa (OTU > 0.001) based on a correlation analysis within ecosystems **(A)**. The networks represent relationships between co-occurring ecosystems and the edges represent co-occurrence relationships consistent at the 0.6 correlation level. The nodes represent microbial eukaryotic taxa. Maximum-likelihood phylogenetic trees constructed with OTUs (relative abundance > 0.1) from module 5 for potentially parasitic Syndiniales and Stramenopiles **(B)**, and from module 6 for Spumellarida **(C)**. The size of bubbles reflects the proportion of each taxon.

To elucidate the interactions between the microbial eukaryotic groups in different environments, network analyses were conducted based on the OTUs selected with a relative abundance greater than 0.001 (Supplementary Table 1). The resulting network consisted of 549 nodes and 2,375 edges, and the modularity index was 0.76. As the index was > 0.4, this suggests that the network had a modular structure (Newman, 2006; Figure 6A). In total, six major modules with a correlation of 0.60 were defined, and these were composed mainly of the SAR super-group.

The phylogeny of the typical major OTUs (relative abundance > 0.1) in Modules 5 (Figure 6B) and 6 (Figure 6C) that were also analyzed by constructing maximum-likelihood phylogenetic trees. The correlations found between the Syndiniales (mainly Dino-Group-I, Dino-Group-II) and MAST-9 in modules 5, Syndiniales and Radiolaria in modules 6 might suggest that they have a potential parasitic relationship. The OTUs in Module 5 were phylogenetically affiliated with Syndiniales (mainly Dino-Group-I and Dino-Group-II) and MAST-9 clusters, and were closely linked to phylotypes from the anoxic basin or oxygen deficient zones. All OTUs affiliated to Dino-Group-II had highest relative abundance at Site F (0.49–3.21%, mean = 0.82%), while there was no obvious regional difference for Dino-Group-I and MAST-9. In addition, OTUs in Module 6 were affiliated with the Spumellarida cluster, containing phylotypes from bathypelagic waters and anoxic basins. The lowest relative abundance of Spumellarida was occurred at Site F (0.10–1.31%, mean = 0.42%). In contrast, OTUs in Module 4 shared higher similarities with sequences from other extreme habitats, such as hydrothermal sediments, high-Arctic fjords, and sea-ice edges.

## Discussion

The role of microbial eukaryotes as key components of the microbial loop has not been well studied in the deep-sea cold seeps. Numerous parasitic ecological types have been reported in the chemosynthetic hydrothermal vents (Moreira and López-Garcia, 2003) and anoxic marine environments (Torres-Beltrán et al., 2018). Diversity and distribution of microbial eukaryotes in the cold seeps, as a typical deep-sea chemical chemosynthetic ecosystem, have been rarely studied (Pasulka et al., 2016), and the potential trophic relationships of microbial eukaryotes in this environment have not been explored. In the current study, parasitic groups were predominant in all samples, although taxa associated with heterotrophic and symbiotic species were found as well. This suggest that parasitism maybe prevalent in deep-sea chemosynthetic habitats.

### Spatial Variations

The closely clustered microbial eukaryotic communities in the two cold seeps were distinct from those in the trough, indicating that the composition of the microbial eukaryotic communities cannot simply be explained by geographical proximity. In the cruise, *in situ* chemical profiles (e.g., sulfides, oxygen, and methane) were recorded during each sampling to confirm the occurrence of the cold seeps and guarantee that samples were collected within the range of cold seeps. The significant correlations between certain groups of microbial eukaryotes and nutrients of the sediments revealed in this study could be attributed to the depth effect. Previous study conducted correction analysis between physic-chemical parameters and microbial eukaryotes in the waters and sediments of cold seeps, and found only depth had significant effect on the distribution of microbial groups, although the influence of chemical parameters might not be excluded (Pasulka et al., 2016).

The discrepancy between the cold seeps and trough could be explained by those “indicative OTUs” belonging to Alveolata (mainly Dino-Group-I and Dino-Group-II) and Rhizaria (mainly Cercozoa and Spumellarida) generated from a SIMPER analysis. The predominance of the SAR super-group in the cold seep samples collected here is consistent with their known wide distribution, being especially predominant in the water and sediments of coastal and open oceanic regions (Countway et al., 2007; de Vargas et al., 2015; Pernice et al., 2015). The fact that Stramenopiles were more abundant in the deepest sediment samples with a significant negative correlation with the total carbon content measured might be due to their key role in the overall function of marine ecosystems as a means for transferring large amounts of organic carbon into the upper trophic levels and controlling the population size of prokaryotes (Logares et al., 2014). Here, the predominance of Alveolata in all the sediments (i.e., both of the cold seeps and the trough) is consistent with the findings of previous studies conducted on submarine caldera floor and Southeast China sediment regions (Takishita et al., 2005; Gu et al., 2020). Radiolaria and Cercozoa, as the two major groups of Rhizaria in our samples, is also consistent with previous reports. For example, Radiolaria were shown to be predominant in 88 surface sediment samples collected over a broad area of the southwest Pacific/Southern Ocean (Cortese and Prebble, 2015). In addition, Cercozoa have been recognized to be major components of predatory protozoa (Fiore-Donno et al., 2019), which frequently dwell in marine sediments of the deep-sea floor (Pawlowski et al., 2011; Wu and Huang, 2019). Typical phototrophic planktons, Bacillariophyta, Dictyochophyta, Prasinophyceae, and Pelagophyceae, were found here in sediments of cold seeps and trough, and they were very likely originated from sinking particles from above water column (Pernice et al., 2016). This suggests that deep-sea sediments are a reservoir of eukaryotic DNA, which contained not only the endemic eukaryotic fauna but the DNA originated from the planktonic taxa, cysts and spores as well (Pawlowski et al., 2011). Future studies at RNA levels together with cultivation and microscopy observation would help to reveal those metabolically active groups.

An obvious spatial distribution of certain taxonomic groups along the vertical sediment profiles was demonstrated. Indeed, with regards to Rhizaia, Radiolaria were more abundant in the middle or deep sediment layers, this might be attributed at least in part to their siliceous skeleton, which can withstand the higher pressures of the deeper layers (Boltovskoy, 2017). In contrast, Cercozoa dominated the surface or middle sediments, consistenting with a previous study conducted in the SCS (Wu and Huang, 2019). Low abundance of heterotrophic Stramenopiles in our sediment samples was perhaps due to the concomitant low abundance of prokaryotes that they feed on (Burki and Keeling, 2014). Because prokaryotic cell abundance in the sediments of cold seeps ranged from 10^5^ to 10^6^ cells/g sediment (wet weight), which was significantly lower than those detected from the adjacent abyssal plain in the SCS (Zhang et al., 2012). Microbial eukaryotes have a wide distribution. The major groups revealed from this study have been reported previously from deep-sea methane seeps (Pasulka et al., 2016) and hydrothermal vents (Moreira and López-Garcia, 2003), thus further strengthening the connection between microbial eukaryotic composition and deep-sea chemosynthetic ecosystems with similar environmental features, such as high hydrostatic pressure, presence of reductive gases, and potentially other driving force yet to be identified.

### Rare and Abundant Microeukaryotes

Previous studies on oceanic microbial communities have largely focused on the most abundant taxa. However, the diversity of microbial communities can better be explained by identifying the low-abundant or rare taxa (Galand et al., 2009). In our study, the community biogeographic patterns, which were based on the intermediate OTU category, were similar to those based on the total number of OTUs. This might be because the intermediate category contained the largest number of OTUs. Therefore, this category, rather than the excessive shared and specific OTUs contained in the abundant and rare categories, respectively, is a better representation of the whole microbial eukaryotic community. The shared OTUs differed among the abundant, intermediate, and rare OTU categories, and not all the abundant taxa (i.e., 171 OTUs) were shared by all the samples. This might be related to both geographic distance and environmental selection. Moreover, the rare taxa might be more susceptible to stochastic processes, such as ecological and evolutionary drift (Hanson et al., 2012; Zhou et al., 2014), and therefore might act as a means for maintaining the community diversity and keep the balance and resilience of different ecosystems.

### Parasitic Relationship in Cold Seeps

Parasitism is an efficient strategy that facilitates microbial eukaryotes for constant access to higher concentrations of organic materials (Worden et al., 2015). Parasitism is widespread in the extreme marine environments (Torres-Beltrán et al., 2018) and hydrothermal vents, and likely contributes carbon turnover in deep-sea food webs (Moreira and López-Garcia, 2003). Parasites could affect a number of trophic links and render the natural food web organization and the genetic composition of natural communities more complex (Lafferty and Kuris, 2002). Therefore, the ecological significance of the prevalence of parasitism in the deep-sea chemosynthetic environments deserve further investigations.

A co-occurrence analysis was conducted to explore the potential ecological relationship among different microbial eukaryotic groups. Results of this analysis showed that Syndiniales were strongly linked to MAST-9 (Module 5; Figure 6B) and Radiolaria (Module 6; Figure 6C). Because of the lifestyle of several Syndiniales species (Giovannoni and Stingl, 2005; Chambouvet et al., 2011), they are generally believed to be parasitic and can infect a wide variety of free-living organisms such as other dinoflagellates and Radiolaria (Giovannoni and Stingl, 2005; Bochdansky et al., 2010). Potential hosts include other Syndiniales, Dinoflagellates, ciliates, Rhizarians, and Metazoans (Guillou et al., 2008; Bråte et al., 2012). Maxillopoda, Oedaleus, and Hydrozoa have also been reported as the major groups of metazoans that from parasitic relationship with Syndiniales (Torres-Beltrán et al., 2018). However, those metazoans were not found in our study (Supplementary Figure 1), implying that protist are the major hosts associated with the parasitic Syndiniales at cold seeps.

Syndiniales displayed a high level of diversity, which might contribute to their successful colonization over a wide range of ecological niches (Guillou et al., 2008). It was not surprising to find clade 1 and 2 of the Dino-group-I, affiliated to the parasitic Syndiniales, had higher relative abundance in this study (Supplementary Figure 2). Group I was specialized in the anoxic environments, particularly sediments (Guillou et al., 2008), and these two clades had the capability to infect radiolarian Spumellarida (Dolven et al., 2007). Dino-group-II, which are synonymous with amoebophrya (Guillou et al., 2008), was dominated by clade 15 (Supplementary Figure 2). This clade was retrieved from the deep-sea cold seeps (Takishita et al., 2007) infecting a broad range of marine dinoflagellates (Cai et al., 2020).

The strong correlations found between the Syndiniales and Radiolaria might suggest that they have a parasitic relationship. Radiolaria act as hosts for many living microorganisms, such as Prymnesiophyceae, Prasinophyceae, and dinoflagellates (Decelle et al., 2012) including Syndiniales (Bråte et al., 2012). It is well known that Radiolaria are one of the major players that export organic carbon to the deep sea (Countway et al., 2007; Schnetzer et al., 2011). A parasitic relationship between Syndiniales and Radiolaria may play a crucial role in helping to maintain the microbial ecosystems of the deep-sea cold seeps in the SCS.

Results of this study indicate that Spumellarida was affiliated to Polycystinea. This is similar to sequences found in bathypelagic waters (Xu et al., 2016) and an anoxic basin (Edgcomb V. et al., 2011). Spumellarida has previously been reported to be a micro-planktonic Protozoa with parasitic strategy (Yuasa et al., 2012), and so it might exist in both deep waters and surface sediments, and have a parasitic lifestyle in both the cold seeps and troughs of the SCS. The presence of MAST-9 and Spumellarida in the sediment samples of our study might be favored by the environmental conditions.

MAST-9 (marine stramenopile 9) also exhibited strong correlations with Syndiniales. Most of the MAST-9 were phylogenetically affiliated to those from an anoxic basin or oxygen deficient zone (Figure 6B; Takishita et al., 2005, 2007). MAST-9-related lineages are treated as specialized groups, well adapted to anoxic or suboxic habitats (Massana et al., 2004b; Torres-Beltrán et al., 2018), and have been retrieved from hydrothermal vents (Takishita et al., 2005) and deep-sea methane cold seeps (Takishita et al., 2007) as well. Results of this study suggested that members of this group might have a parasitic relationship with Syndiniales at cold seeps.

## Conclusion

As our current study was based solely on 18S rRNA gene sequences, future additional information generated through microscopic observation and taxonomy identification with higher resolution will help us to gain a deeper understanding of the specialization and potential trophic relationships of microbial eukaryotic groups in different ecological niches of deep-sea ecosystems. If additional sample material was available, specific marker genes such as the oligonucleotide probes for Group II Syndiniales (Chambouvet et al., 2008) could be applied to gain additional evidence of active parasitism in the sediments in the future study.

## Data Availability Statement

The datasets presented in this study can be found in online repositories. The names of the repository/repositories and accession number(s) can be found below: https://www.ncbi.nlm.nih.gov/, PRJNA742151.

## Author Contributions

YZ analyzed data and wrote the first draft. NH performed the experiment and analyzed the data. MW and HL revised the manuscript. HJ collected the samples and designed the research. All authors contributed to the article and approved the submitted version.

## Conflict of Interest

The authors declare that the research was conducted in the absence of any commercial or financial relationships that could be construed as a potential conflict of interest.

## Publisher’s Note

All claims expressed in this article are solely those of the authors and do not necessarily represent those of their affiliated organizations, or those of the publisher, the editors and the reviewers. Any product that may be evaluated in this article, or claim that may be made by its manufacturer, is not guaranteed or endorsed by the publisher.

## Footnotes

1
www.novogene.com
