# Supplementary material for: Microbial Eukaryotes Associated With Sediments in Deep-Sea Cold Seeps
Source: Front Microbiol. 2021 Dec 22;12:782004. doi: 10.3389/fmicb.2021.782004 (PMC8740301; doi:10.3389/fmicb.2021.782004)
Supplement: Supplementary file 2 [file Presentation_1.pptx]

## Slide 1
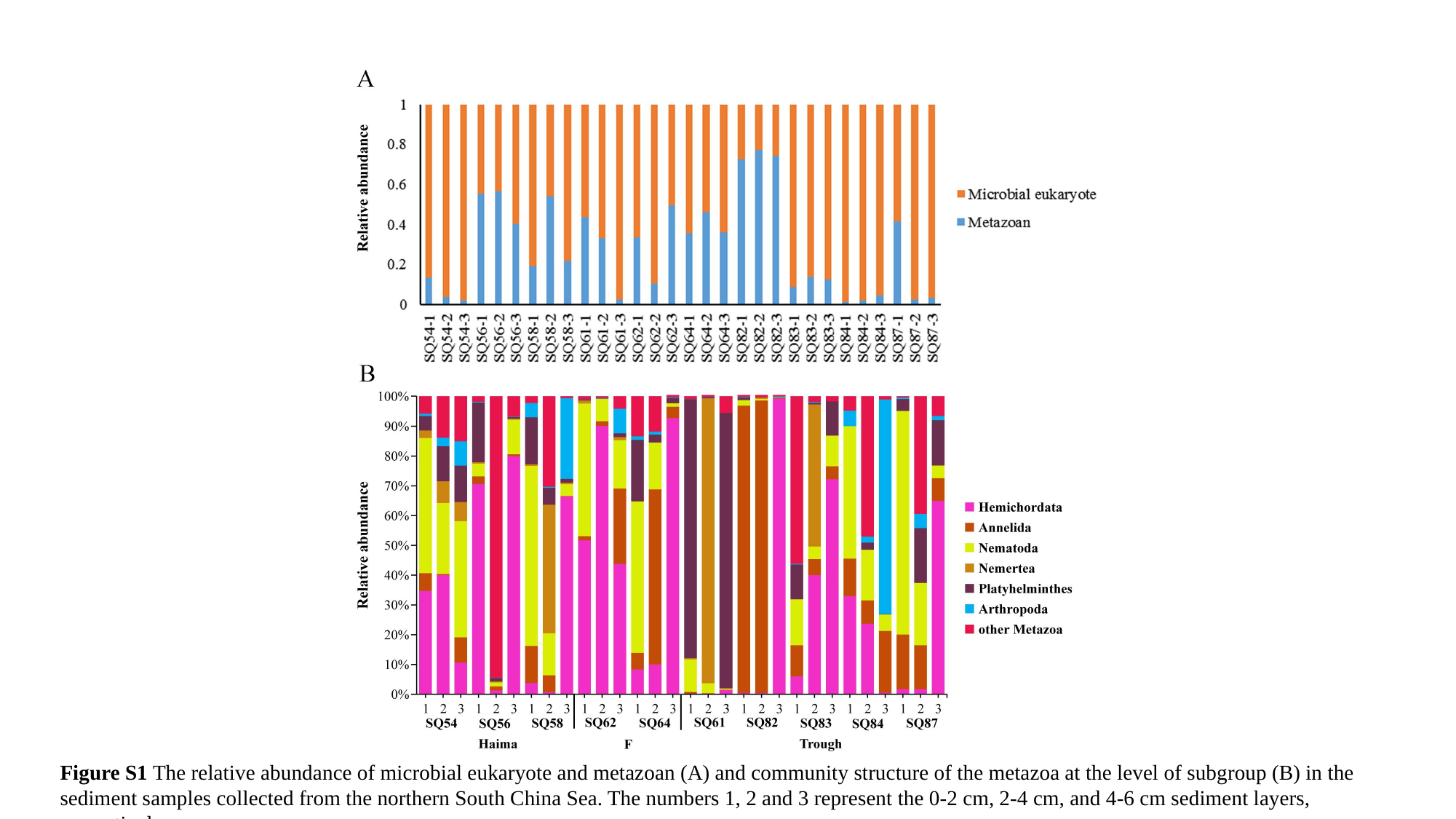

Figure S1 The relative abundance of microbial eukaryote and metazoan (A) and community structure of the metazoa at the level of subgroup (B) in the sediment samples collected from the northern South China Sea. The numbers 1, 2 and 3 represent the 0-2 cm, 2-4 cm, and 4-6 cm sediment layers, respectively.

## Slide 2
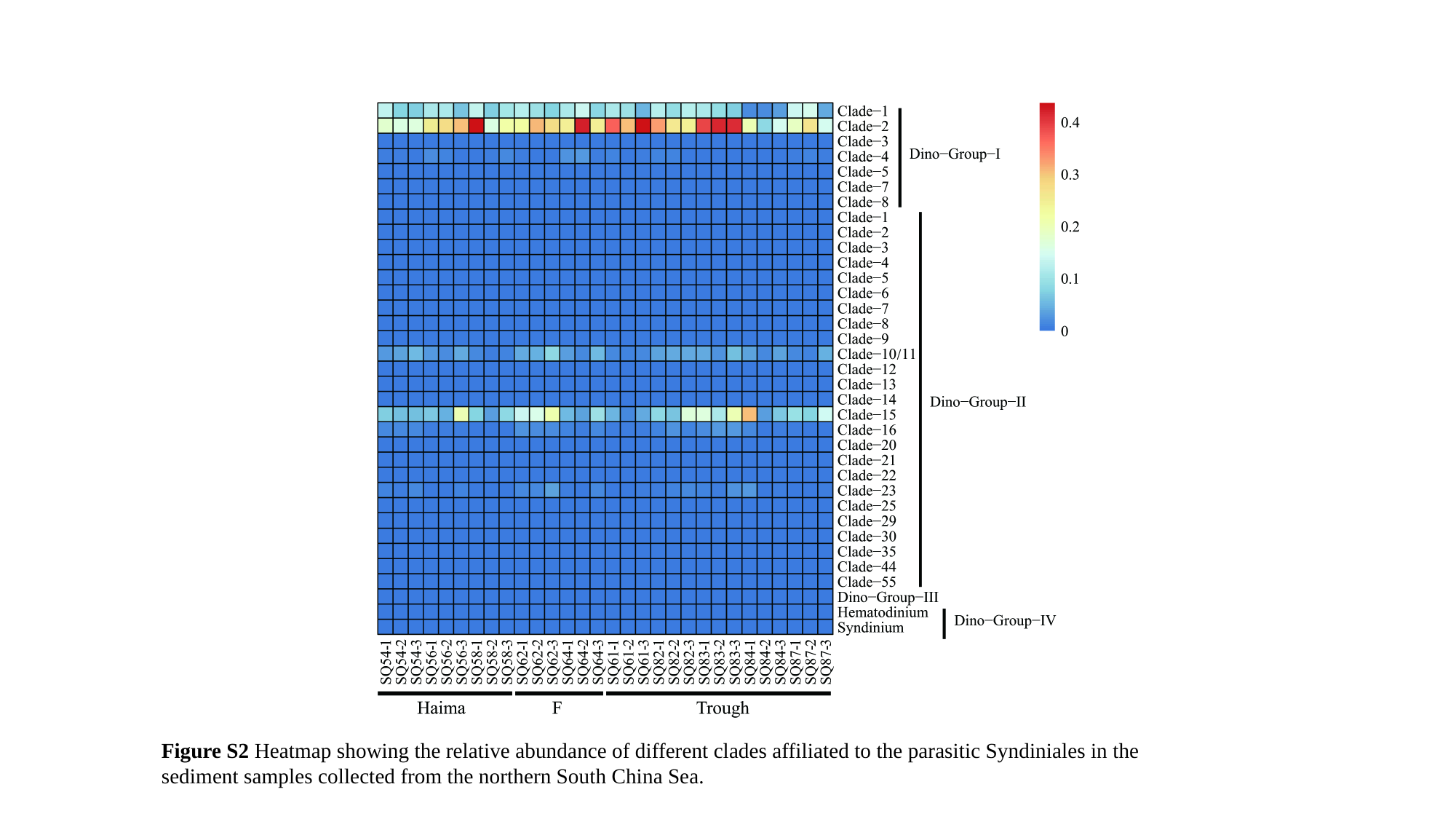

Figure S2 Heatmap showing the relative abundance of different clades affiliated to the parasitic Syndiniales in the sediment samples collected from the northern South China Sea.
